# Recommendations for the Implementation of Hospital Based HTA in Poland: Lessons Learned From International Experience

**DOI:** 10.3389/fphar.2020.594644

**Published:** 2021-05-13

**Authors:** Małgorzata Gałązka-Sobotka, Iwona Kowalska-Bobko, Krzysztof Lach, Aneta Mela, Maciej Furman, Iga Lipska

**Affiliations:** ^1^ Institute of Healthcare Management, Lazarski University, Warsaw, Poland; ^2^ Institute of Public Health, Faculty of Health Science, Jagiellonian University Medical College, Krakow, Poland; ^3^ National Institute of Cardiology (Poland), Warsaw, Poland; ^4^ Department of Experimental and Clinical Pharmacology, Medical University of Warsaw, Warsaw, Poland; ^5^ National Health Fund, Warsaw, Poland

**Keywords:** decision making, hospital management, health technology assessment, hospital based health technology assessment, innovative medical technologies

## Abstract

**Introduction:** The main challenge of modern hospitals is purchasing medical technologies. Hospital-based health technology assessments (HB-HTAs) are used in healthcare facilities around the world to support management boards in providing relevant technologies for patients.

**Aim:** This study was undertaken to update the existing body of knowledge on the characteristics of HB-HTA systems/models in the selected European countries. Insights gained from this study were used to provide an optimal approach for implementing HB-HTA in Poland.

**Materials and methods:** Firstly, we carried out a systematic review in PubMed and embase. Secondly, we searched for gray literature via the AdHopHTA online handbook and the design book of the AdHopHTA project, as well as literature describing healthcare systems provided by the WHO. Then, we conducted in-depth interviews with HB-HTA experts from four countries. Finally, we selected ten countries from Europe and prepared frameworks for data collection and analyses.

**Results:** The selected countries (Switzerland, Spain, France, Italy, Denmark, Finland, Sweden, the Netherlands, and Austria) are examples of decentralized or deconcentrated healthcare systems. In terms of HB-HTA, differences in organisational models (independent group, stand-alone, integrated-essential, integrated-specialised), type of financing (internally vs. externally), collaboration with an HTA National Agency and other stakeholders (e.g., Patients’ Associations) were identified. HB-HTA engages multi-skilled staff with various academic backgrounds and operates mainly on a voluntary basis.

**Conclusion:** Strengths and weaknesses associated with various organisational models must be carefully considered in the context of support for decentralized or centralized models of implementation while embarking on HTA activities in Polish hospitals.

## Introduction

Health Technology Assessment (HTA) influences drug reimbursement decisions are made by respective bodies on the basis of reliable scientific research results from the perspective of a particular healthcare system (INAHTA, 2015). An EU health technology assessment organization network EUNetHTA explains that HTA is a “multidisciplinary process that summarizes information on medical, social, economic, and ethical issues related to the use of a given health technology in a systematic, transparent, impartial and robust manner. Its purpose is to provide the information needed to create safe and effective patient-centred health policies and a desire to achieve the best value” (EUnetHTA, 2015).

Currently, HTA is an internationally recognized and widely used approach in, e.g., informing decisions on drug reimbursement and pricing. In the European Union, such decisions following the assessment of health technologies are strictly national. Member states independently and separately create their own HTA frameworks with the establishment of a local HTA agency (Włodarczyk et al., 2012). The main purpose of these agencies is to support healthcare system stakeholders in making decisions on optimal resource allocation.

In 2005, Poland established the Agency for Health Technology Assessment, which serves the Minister of Health as an advisory body by giving recommendations on whether health technologies should be financed (Gulácsi et al., 2014). Since 2012, Poland has been an example of a country where no medicine is reimbursed without formal procedures (Barnieh et al., 2014). HTA is an important and formal part of the decision-making process regarding the reimbursement and pricing of new pharmaceuticals in Poland (Lipska et al., 2017).

HTA does not only refer to medicinal products or a function within national health technology assessment agencies. Usually, healthcare facilities, such as hospitals, need a more practical and contextualized assessment related to the use of a specific clinical procedure, medical device, or equipment in their own settings (Sampietro-Colom, 2012) Hence, a hospital-based health technology assessment (HB-HTA) process is conducted, which applies tools used in HTA processes in the context of individual hospitals. Key reasons for adopting HTA at the hospital level are threefold: 1) new challenges (e.g., socioeconomic) have caused changes in purchasing innovative technologies; 2) hospitals need to stay abreast of new, innovative technologies for which an assessment from a national HTA agency is not yet available; 3) the efficient management of limited resources by hospitals is vital. The major disparities between the traditional HTA at the national/regional level and at the hospital level are: 1) the context (country/region vs. hospital); 2) informational needs (e.g., a budget impact analysis is far more important than a cost-effectiveness analysis at the hospital level, and also strategic aspects are a newly-identified assessment domain important at the hospital level); 3) the types of technologies evaluated (assessments of non-drug technologies are conducted more frequently at the hospital level); and 4) need for a timely assessment associated with a scope of the assessment (a comprehensive HTA is usually conducted at the national level, which takes from one to even two years, while at the hospital level, a mini-HTA is carried out with a usual delivery time of three months) (Sampietro-Colom et al., 2015). However, both HB-HTA and national/regional HTA pursue the same goal, that is, to inform decision-making on the optimal resource allocation with regards to investment in health technologies.

Technology assessment units in hospitals are currently present on all continents: from European countries (such as Spain, Italy, Denmark, and Estonia) (Sampietro-Colom and Martin, 2017), North America (the United States and Canada), South America (Brazil and Argentina), to Africa (South Africa), Australia, and New Zealand (Sampietro-Colom and Martin, 2017). An EU-funded project, AdHopHTA (“Adopting hospital-based HTA in the EU”), and other initiatives comprehensively characterized technology assessment units from all around the world (Sampietro-Colom et al., 2015; Sampietro-Colom and Martin, 2017). However, several years have passed since those were published.

Currently, there is no HTA activity in Polish hospitals that the authors are aware of and since the most recent scientific contribution characterizing HB-HTA worldwide (Sampietro-Colom et al., 2015) was published in 2016, our study was designed to update the knowledge in the field and, based on state-of-art knowledge on HB-HTA, lay grounds for informing the optimal model of HB-HTA to be implemented in Poland.

This study was conducted as part of the ongoing research and implementation of the project “HB-HTA-PL” funded by the Polish National Center for Research and Development (2019–2021).

## Aim of This Study

The study was undertaken to update the existing body of knowledge on the characteristics of HB-HTA systems/models in selected European countries. Insights gleaned from this study will be used to inform an optimal approach while implementing HB-HTA in Poland. This study was conducted as part of the ongoing research and implementation of the project “HB-HTA-PL” funded by the Polish National Center for Research and Development (2019–2021) (HB-HTA, 2020).

## Materials and Methods

First, a literature review was conducted to identify developed HB-HTA systems from different regions of Europe, with decentralized hospital management, and with a similar gross domestic product. The following countries were considered for analysis: Switzerland, Spain, France, Italy, Denmark, Finland, Sweden, the Netherlands, and Austria.

The following sources of information were utilized in this study:

### A Review of Literature in Medical Databases and Gray Literature

A review of literature in Medline (via PubMed), and embase was carried out. Two search strategies were applied (Supplementary Appendix S1) with a cut-off date of December 29, 2019. The first, more specific, referring only to hospital based HTA search terms, and the second, more sensitive, referring to broadly understood collaboration activities by HTA units/organisations. A time restriction of the last 10 years was applied to capture the most recent developments in the field. Detailed information on the keywords used within the search strategies and the study flowchart based on two search strategies in the Medline and embase database (according to the PRISMA statement) are presented in Supplementary Appendices S1, S2 (Supplementary Tables S5–S8 and Supplementary Figures S1, S2).

Additionally, as part of the review, gray literature sources were screened: the AdHopHTA handbook: Hospital-based Health Technology Assessment, The Next Frontier for Health Technology Assessment book: and the WHO Health Systems in Transition database. In the latter, a review of the WHO European Observatory on Health Systems and Policies database, the Health Care Systems in Transition series, was conducted to identify features of the health system models important in the context of HB-HTA development.

Any publication that described the experience or organisational model of a single HB-HTA/unit at the regional/national level or collaboration practices/interactions between HB-HTA units or collaboration practices/interactions between HB-HTA units and national/regional HTA agencies in the selected European countries were included in the review. Conversely, any publication that characterized national/regional HTA agencies or described any form of collaboration practices/interaction between HTA agencies at the regional/national level were excluded.

### In-Depth Expert Interviews

Following the literature review, semi-structured telephone interviews with HB-HTA experts were conducted to address gaps identified in the secondary research as well as to corroborate the findings from the literature review. Four interviews were conducted with HB-HTA experts from Austria, Spain, France, and Denmark. A short questionnaire consisting of five open-ended questions was prepared and sent to the experts ahead of the interviews as stimuli for the conversation:

1. What has changed over the last 5 years in terms of collaboration practices/interactions between your HB-HTA unit and other units/regional or national HTA agencies?
2. Have additional standards or formalization been developed for HB-HTA in your country?
3. Have new stakeholders relevant from the perspective of HB-HTA appeared? If so, are they a driving force or a barrier to HB-HTA development?
4. What are the major advantages and disadvantages of the HB-HTA model in your country?
5. What is the direction of HB-HTA development and networks created at the domestic and international levels for the future?

Each interview was commenced by providing the background for the research as well as the summary of findings from the conducted literature review. Interviews were prepared by two researchers (IKB, KL) and conducted by one researcher (KL) in October 2019. Interview recordings were used to prepare written transcripts and then analyzed by three researchers (IKB, AM, MF).

### Data Extraction and Analysis

Data from the included articles were extracted using a pre-designed analytical framework (designed by KL), whose main purpose was to capture data on the general characteristics of the HB-HTA unit, including its model (according to the nomenclature developed by AdHopHTA researchers: independent group model, integrated-essential—HB-HTA model, stand-alone—HB-HTA units, integrated-specialized HB-HTA units) (Sampietro-Colom and Martin, 2017), including interactions between HTA units in hospitals, and taking into account any collaboration practices with national/regional HTA entities or other entities (Table 1). Pilot extractions were conducted by two researchers (AM, MF) and then checked independently by another researcher (IKB) for accuracy.

**TABLE 1:** Analytical framework for data extractions.

Analytical frame	Scope
HB-HTA unit organisational model	•Independent group
	•Integrated-essential HB-HTA
	•Stand-alone HB-HTA unit
	•Integrated specialized HB-HTA unit
Characteristics of HB-HTA unit and its interaction with other stakeholders	•Description of activities/interactions
Level of interaction with other stakeholders	•Internal (inside one country)
	•External (between countries)
Interaction Type	•Informal
	•Formal
	•Voluntary
	•Mandatory
Stakeholders involved in the interactions	•HB-HTA organisational level (stakeholders from various governance and management level, e.g., self-governmental bodies)
	•Additional important actors involved and their role
Additional information	•All other relevant details

Source: Own study based on AdHopHTA Handbook.

## Results

30 publications included in the review provided the most recent characterization of HB-HTA units in the selected European countries. The nine countries, for which information on HB-HTA activity was available, have a decentralized hospital management system, a similar gross domestic product, and established HB-HTA units at select hospitals. In addition to HB-HTA units, national HTA agencies (if any) of the select countries were also characterized in terms of their scope of responsibilities. A detailed description of HB-HTA units and national HTA agencies are shown in Supplementary Appendices S3, S4. An analysis of the hospital sector across European countries by (Chevalier et al., 2009) and Kowalska-Bobko, I. provides comparable data on hospital governance. Based on the analysis, hospital management systems have been derived in terms of decentralization, centralization, and “deconcentration”. It can be concluded from literature and the conducted research, that decentralization of the health system supports hospital efficiency. The concept of decentralization means the transfer of power from the state level to the level of autonomous territorial self-governments. Decentralization includes a wide range of scenarios in the EU, with varying degrees as the transfer of powers to elected infra-national bodies. Meanwhile, deconcentration, means a transfer of power from the central level to separate government institutions operating at the regional or local level (Kowalska-Bobko, 2017). Hospital management is decentralized in Belgium, Austria, Germany, Denmark, Finland, Sweden, Spain, Italy, as well as in several Central-Eastern European countries by self-government hospital ownership. Bulgaria, France, Greece and Portugal are, in turn, the countries characterized by a “deconcentrated” hospital management system where central government bodies organize inpatient care (Table 2). In Poland, the process of decentralization in health care was initiated after the fall of the communist regime and finally consolidated after the separation of three levels of local government—commune in 1990, county and regional self-government in 1999. Municipalities are responsible for primary health care, and counties and regional self-government for hospitals. The regional self-government is responsible also for strategic planning in health care for the benefit of its population, while the governmental deconcentrated regional authorities are responsible for mapping health needs (Sowada et al., 2019).

**TABLE 2:** Selected countries for the analysis of HB-HTA models.

Country	GDP per capita (2018) [PPP, USD] (List of Countries by Projected GDP, 2018)^a^	Type of healthcare system
Switzerland	68,060	Decentralized
Spain	39,715	Decentralized
France	45,342	Deconcentrated
Italy	41,830	Decentralized
Denmark	55,671	Decentralized
Finland	46,735	Decentralized
Sweden	53,208	Decentralized
The Netherlands	56,328	Decentralized
Austria	54,454	Decentralized

a GDP per capita expressed taking into account the purchasing power parity correction (PPP).

Source: Own study based on https://data.worldbank.org/indicator/NY.GDP.PCAP.PP.CD?locations=AT-ES-NL-FR-SE-FI-DK-IT-CH.

A detailed characterization of each health care system in which HB-HTA is organized within select hospitals is provided below.

### Results

Our review identified differences between the current HB-HTA units in nine European countries. Although healthcare systems are significantly developed in all analyzed countries, they represent various approaches to HB-HTA. They differ in terms of the HTA bodies’ structure, HB-HTA staff qualifications, and the role of hospital boards in the decision-making process, as well as presenting different HB-HTA models (Sampietro-Colom et al., 2015; Martelli et al., 2016).

The countries selected in our analysis of the hospital health technology assessment system have similar GDPs and a decentralized approach to health care management (Sampietro-Colom et al., 2012). The characteristics of the analyzed countries in terms of similarities and differences of HB-HTA systems have been presented below;

HB-HTA can be analyzed and modeled with respect to:

a. Micro-perspective—organizational practices within HB-HTA units and their level of formalization, i.e., from independent, less formal teams to specialized HB-HTA units.
b. Macro-perspective—various complex relationships in a health care system and how they can support the creation of HB-HTA units (Sampietro-Colom et al., 2012).

While micro-perspective has already been discussed by AdHopHTA researchers (Sampietro-Colom et al., 2015), we undertook a broader consideration of macro-perspective of HB-HTA to inform the implementation of HTA at Polish hospitals.

In the micro-perspective, a key aspect is the level of independence of HTA units in hospitals. HB-HTA units to better organized have to consider different factors:

• Size of the unit;
• Level of development and staff experience in HB-HTA;
• Unit’s position with relation to other stakeholders.

The characteristics that best define an HB-HTA unit are the following: 1. Formalization. Procedures and rules organizing HB-HTA unit’s day to day operations (standard operating procedures and formal documents/guidance). 2. Specialization. This means the extent to which duties and tasks are fragmented into independent roles in the HB-HTA unit. A highly specialized unit is one that can manage various types of HTA processes dedicating specific resources to those processes such as a project team, working on scientific grants, full-time employers and/or specific formal procedures, for example, specific procedures on each type of health technology to be assessed. 3. Integration. This holds for the level of common work between the HB-HTA unit and other healthcare stakeholders inside or out of the hospital. Integration is high if the HB-HTA unit has multiple networks with other actors that are conducting HTA at other institutional levels (i.e., at national or regional levels, the public payer, or universities). 4. Authority and centralization of power. This refers to the authority to take decisions within the HB-HTA unit. When a decision is to be taken, if it is delegated to lower organisational levels in the HB-HTA unit (e.g., to the person responsible for an HTA project), that unit is considered decentralized. Decentralization is represented when a single clinician is responsible for recommending the technology to be used within a hospital. 5. Professionalization. This refers to the degree of expertize available or training undergone by the HB-HTA unit’s staff (their knowledge, experience and education) (Sampietro-Colom et al., 2015).

Three of these variables—1) specialization, 2) formalization, and 3) the level of integration usually defines the organizational arrangements of HB-HTA units. Highly specialized units are more formal because their existence is based on legal regulation. Usually HB-HTA units that existed for a long period of time are more formal and highly specialized. On the other hand, some of the less “mature” HB-HTA units prefer to maintain flexibility, being less specialized, structured and formalized. Integration with other organizations that are conduct in HTA can be based on formal agreements or informal collaborations.

A summary of the analyzed countries with a focus on similarities and differences of HB-HTA systems is presented in Table 3. HB-HTA units in the analyzed countries have different organizational models based on their respective needs. Some relationship can be observed between the choice of organizational model, its financing, and the existence of a national HTA agency. An integrated-specialized model with external funding seems to be more frequently present for HB-HTA units in those countries where a national HTA agency exists (France, Finland, Sweden, and the Netherlands) (Jivegard and Ektroth, 2007; Halmesmäki et al., 2016; Martelli, 2019) although in several countries the sources of HB-HTA unit funding is both internal and external (France, the Netherlands) (Bos, 1995; AGENAS, 2018). Integrated-specialized HTA units at hospitals also more often tend to interact with the payer/insurer or the Ministry of Health (France, Denmark, and the Netherlands) (Kidholm et al., 2009; Lennart et al., 2017). Regardless of the organizational model, most of analyzed HB-HTA units have some level of informal interaction with regional governments or hospital district representatives.

**TABLE 3:** Characteristics of the analyzed countries in terms of similarities and differences of HB-HTA systems.

Country/ Category	National HTA agency	HB-HTA model				Source of financing		Stakeholders		
	Typical HTA Agency at the national level	Independent group^a^	Stand-alone^b^	Integrated-essential^c^	Integrated-specialised^d^	Internally (from hospital funds)	Externally (scientific grants and regional funds)	National HTA Agency or equivalent	Payer/insurer or Ministry of Health	Regional governments (hospital district representatives)
Switzerland—CHUV	—			✓		✓		✓		
Switzerland—HUG		✓				✓				
Switzerland—EHNV		✓				✓		✓		
Spain—HCB	—		✓			✓				✓
Spain—VR&VM				✓		✓				✓
Spain—HStJD		✓				✓				✓
France	✓				✓	✓	✓	✓	✓	✓
Italy	✓		✓			✓	✓		✓	
Denmark	—				✓	✓	✓		✓	
Finland	✓				✓		✓	✓		✓
Sweden	✓				✓		✓			✓
The Netherlands	✓				✓	✓	✓		✓	
Austria	✓	✓					✓	✓	✓	✓

a Internal hospital units operating as an “independent group” that informally supports managerial decisions on health technologies.

b With highly specialized and formalized units within hospitals, operating without strong influences from other external stakeholders, such as national HTA agencies (currently the most frequent model in Europe).

c Units with limited staff but capable of involving other stakeholders acting as allies in their HTA activities.

d Units influenced by formal collaboration with the national or regional HTA agencies. Generally, the involvement of HB-HTA units in the technology adoption process is considered good practice and the HTA-based recommendations are closely followed by hospital decision-makers.

CHUV, Lausanne University Hospital; HUG, Geneva University Hospital; EHNV, The North Vaudois Hospital; HCB, The Hospital Clinic of Barcelona; VR&VM, the Virgen del Rocio and Virgen de la Macarena hospitals; HStDJ, the Hospital Sant Joan de Deu.

From the macro-perspective, undoubtedly, the common feature of the countries analyzed is the understanding of the need for networking between HB-HTA and other stakeholders in healthcare. Among the countries analyzed, the involvement of both central institutions, such as the public payer or the national/regional HTA agency can clearly be seen. Representatives of hospital districts, i.e. institutions connecting regional healthcare providers, also play an important role. There is much better cooperation among HB-HTA employees in Europe when it is implemented at the regional level. It needs to be highlighted that HB-HTA models need to be adapted to national conditions. This means that all crucial aspects, such as both the external and internal environments, should be considered. Secondly, financing and human resources for the HB-HTA units have to be fixed for this concept to evolve to be more widely implemented into the health system. It should be noted that the HB-HTA system exist in some countries such as Switzerland or Denmark despite the absence of a classic HTA institution at the national or regional level (De Pietro et al., 2015).

The analysis of existing HB-HTA models and practices in European countries lays the groundwork for the identification of country-specific practices and scenarios of how the health care systems in the analyzed countries can be leveraged in implementing and promoting HB-HTA in the Polish context.

The bottom-up model with support from the regional level in the analyzed countries (Spain, Italy, Denmark, and Sweden) has been deemed the most applicable scenario of developing HB-HTA in Poland (Kindholm 2019; Lennart et al., 2017). In this model, hospitals gain autonomy in making investment decisions on health technologies and they receive professional support from regional HTA (Italy, Spain) (Bigorra et al., 2009). This model is characterized by a strong degree of decentralization, wherein regions not only own hospitals, but are also responsible for managing and/or financing healthcare services (Denmark, Sweden) (Lennart et al., 2017). In this case, decentralization supports hospital managers of the HB-HTA process by the regions, which collaborate with hospitals respecting their autonomy in making investment decisions.

A second important scenario, represented by Finland, is an HB-HTA unit independent from central institutions and their regulations and procedures, which may stifle hospitals (Halmesmäki et al., 2016). There are also other possibilities, including a bottom-up initiative featuring large, prominent hospitals as leaders in HB-HTA (Switzerland, France, the Netherlands), or hybrid scenarios, like in Austria (Wild, 2019).

The organization and financing of health care in Poland indicate its centralized characteristics, which is also true in relation to managing hospitals, which are usually owned by a local self-government unit. Poland’s centralized healthcare system is also manifested in the financing of healthcare services by one national payer (the National Health Fund). Given these characteristics, it should be assumed that the most plausible scenarios for implementing HB-HTA would be based on regional or central health care system support. In the former scenario, regional authority at the voivoideship regional level is responsible for hospital care; however, a representative of the central government at the voivoideship level also has a lot of healthcare-related responsibilities (e.g., mapping health needs). At the voivoideship level, there are regional offices of the National Health Fund. In this scenario, the voivoideship authority could play a leading role in regional support of HB-HTA. Nevertheless, the support of other institutions at the regional level would be necessary to successfully implement HB-HTA.

Organizing HB-HTA at the central level is inherently associated with stifling its implementation and limiting its autonomy and the level of latitude in shaping its organization and processes. If implemented in a centralized scenario, HB-HTA at hospitals would need a body that could supervise and monitor HB-HTA and its processes, e.g., the national HTA agency (The Agency for Health Technology Assessment and Tariffs). This is a plausible scenario and its functional effectiveness depends on the relationships between the HTA Agency and hospitals. Respecting HB-HTA’s autonomy and mutual support would foster the implementation of such a HB-HTA model.

Plural or hybrid approaches to implementing an HB-HTA model are characterized by bringing together multiple stakeholders, i.e., governmental bodies, local and regional authorities, a national HTA agency, large hospitals, and professional institutions. Austria is an example of a country that utilizes this approach for HB-HTA processes. Engaging multiple stakeholders could pose a serious challenge in Poland when trying to implement HB-HTA. Respecting bottom-up HB-HTA initiatives, it is paramount to consider the optimal means of supporting the initiatives, taking into consideration existing and yet to be created relationships between stakeholders. Austrian HB-HTA functions on a central and regional level. The pioneer of its implementation was a national agency (since 2020, it is the Austrian Institute for HTA (AIHTA), previously the Ludwig Boltzmann Institute (LBI-HTA)), which cooperated with regions responsible for managing healthcare clinics. The need for short and irregular check-ups within the hospitals was pointed out as a result of this cooperation. It should be highlighted that using various methods of assessing medical technologies, such as clinical efficiency analysis or analysis based on international comparisons, proves that the HB-HTA in this country maintains a high level (Kisser et al., 2016).

## Discussion

It should also be highlighted that since 2016 there have been scarce publications on the experience of implementing HB-HTA methods in Europe. It is also the main limitation for this article as it is not possible to analyze and discuss the practical experience of particular countries in this field. The only publication that was available in the medical information databases (PubMed, embase) is the article presenting the functioning of a HB-HTA facility in Kazakhstan, which was established in 2009 (Avdeyev et al., 2019). This unit contributed to considerable savings in the hospital’s budget by eliminating inefficient technologies. Additionally, it was shown that the introduction of a HB-HTA unit significantly streamlined the decision-making process and hospital management.

From a macro perspective, the analysis results show an increased interest and successful application of HB-HTA methods in countries with decentralized systems of managing hospitals. They point to a high probability of drawing attention to HB-HTA issues in Poland because of the decentralization of hospital management similar to that in Scandinavian countries, even Span or Italy, and the need to include regional authorities in the process of standardizing HB-HTA in Poland. Because of the dedicated HB-HTA project executed in Poland with the National Health Fund as its leader, it is possible that the target HB-HTA model will include the important role of a system payer in the overall process related to the implementation of HB-HTA. After all, hospitals expect the payer to provide bonuses with regard to funding services based on innovative medical technologies. Centralizing the funding for medical services in the Polish healthcare system, similarly to the important system role of the Agency for Health Technology Assessment, favors the establishment of cooperation standards where important institutions (National Health Fund, AOTMiT) bear the responsibility for HB-HTA development in Poland alongside other actors active in the regions and hospitals. Including AOTMiT in the process of HB-HTA management in Poland will open up the possibility of obtaining the essential opinions issued by this institution that would allow for the implementation of a particular innovative medical technologies in the guaranteed services package. It is assumed that a specific central-local, macro-micro perspective for the target HB-HTA model in Poland will be achieved.

The analysis results for the micro perspective show that the greatest opportunities for organisational growth within hospitals are held by the units already established and active in the area of accessing technologies and expanding innovations. The deepest interest in HB-HTA ideas was displayed by Polish clinical and highly specialized hospitals that have just created units responsible for evaluating technologies and expanding innovations and investments within their organisational structures, similarly to the integrated essential HB-HTA units or stand-alone HB HTA units. Establishing and developing such units in Polish hospitals is essential for having qualified staff that would help the management make the right management decisions. Therefore, it is important to recruit employees working in the health system, who will constitute a human resource for the newly established HB-HTA units. They may come from system institutions, such as the National Health Fund or the Agency for Health Technology Assessment and Tariffs, or from health departments in local government units.

Table 4 below outlines the key strengths and weaknesses of HB-HTA organisational models from both the hospital and healthcare system perspectives, providing arguments for an informed decision on the implementation of HB-HTA in Poland.

**TABLE 4:** Strengths and weaknesses of each HB-HTA organisational model in the context of HTA implementation at Polish hospitals.

	Strengths		Weaknesses	
	Hospital perspective	Health care system perspective	Hospital perspective	Health care system perspective
Independent group	•“Pioneers” advocating/ promoting HTA units at hospitals	•Meaning of bottom-up initiatives aligned with internal needs	•Unacknowledged importance of HB-HTA among hospital management	•The absence of activity in HB-HTA
	•“Pioneers” promoting evidence-based medicine approach	•Consideration of managerial effectiveness	•Informal HTA process (high level of hospital latitude)	•Inability to compare practices
			•Low level of engagement by “Pioneers” contingent on competence and time capacity	
			•Bias among clinicians with experience in national HTA	
			•Promoting national HTA at the hospital level by clinicians (losing the hospital perspective)	
Integrated-essential HB-HTA unit	•Initiating lefts of competence in HB-HTA	•Positive pressure on external institutions with lower competency in HB-HTA	•Low general activity level in HB-HTA	•The absence of sufficiently standardized procedures enabling comparisons
	•Synergies in resources and competence boosting the HB-HTA process and decision-making ability	•Initiating networking activity with others, e.g., hospital clinics	•Low level of formalization	•Limited outreach of HB-HTA
	•Promoting hospital managerial effectiveness	•Creating opportunity to compare HB-HTA reports		
Stand-alone HB-HTA unit	•formalization of HB-HTA unit in the organization chart of a hospital	•Bolstering managerial effectiveness of the hospital	•Cost of running an HB-HTA unit	•Good practices limited to a particular hospital without outreach
	•Capabilities in HB-HTA for hospital managers	•Potential promoting criterion for best managerial practices at hospitals	•Limiting autonomy of hospital managers in making investment decisions	•The absence of sufficiently standardized procedures enabling comparisons
	•Center of excellence for developing HB-HTA capabilities for healthcare professionals		•formalization of process adversely impacting the willingness to initiate investments in new technologies	
Integrated-specialized HB-HTA unit	•More structured approach to making investment decisions	•Ability to compare cost-effectiveness of assessed technologies	•Formal established collaboration practices with the national HTA agency	•Integration with national HTA
	•High specialization in assessment domains (e.g., economic evaluation of health technologies)	•Ability to identify good practices in HB-HTA	•High level of formalization in division of work within an HB-HTA unit	•High standardization of HB-HTA methodology and processes
	•Improving the managerial and financial effectiveness of a hospital	•Improving effectiveness of public resource allocation in the hospital sector	•Proliferation of organisational structure	
	•Potential criterion for more favourable tariffs related to healthcare services		•Higher administrative costs	

Authors’ own study based on: Sampietro-Colom, L., Lach, K., Cicchetti, A., Kidholm, K., Pasternack, I., Fure, B., Rosenmöller, M., Wild, C., Kahveci, R., Wasserfallen, J.B., Kiivet, R.A., et al., The AdHopHTA handbook: a handbook of hospital-based Health Technology Assessment (HB-HTA); Public deliverable; The AdHopHTA Project (FP7/2007-13 grant agreement nr 305018); 2015. Available from: http://www. adhophta.eu/handbook. Access online: May 25, 2020. L. Sampietro-Colom, and J. Martin (Eds.), Hospital-Based Health Technology Assessment: The Next Frontier for Health Technology Assessment (pp. 39–44). Springer. https://doi.org/10.1007/978-3-319-39205-9_4.

## Conclusion

HB-HTA units are present in European healthcare systems, which differ in terms of centralization, decentralization, and deconcentration; however, the absence of such initiatives in the older types of centralized healthcare systems are more likely to embark on HB-HTA activity. HTA units and committees in hospitals are characterized by their multi-skilled staff, including medical and other professionals, such as those in public health, health economics, and bioengineering, among others. Our review found that HB-HTA units/committees cooperate with various healthcare system stakeholders, that is, with payers, insurers, national and regional HTA agencies, and respective Ministries of Health. Interactions between stakeholders and HB-HTA units is often informal and voluntary. The funding for HB-HTA units comes from internal hospital funds and/or external scientific grants. Our analysis of strengths and weaknesses associated with various organisational models from the hospital and health care system perspective is expected to provide material input in the debate on the future implementation of HTA in Polish hospitals. These strengths and weaknesses must be carefully considered in the context of support for decentralized or centralized models of implementation while embarking on HTA activities in Polish hospitals.

## References

[B1] AGENAS (2018). AGENAS. National agency for regional health services. Measure to improve 2018. Available at: https://www.agenas.gov.it/images/agenas/Agenzia/brochure_AGENAS_en.pdf (Accessed May 25, 2020).

[B2] Avdeyev A. Tabarov A. Akhetov A. Shanazarov N. Hailey D. Kaptagayeva A. (2019). Hospital-based health technology assessment in Kazakhstan: 3 years' experience of one unit. Int. J. Technol. Assess. Health Care 35 (6), 436–440. 10.1017/S0266462318003744 30829189

[B3] Barnieh L. Manns B. Harris A. Blom M. Donaldson C. Klarenbach S. (2014). A synthesis of drug reimbursement decision-making processes in organisation for economic Co-operation and development countries. Value Health 17 (1), 98–108. 10.1016/j.jval.2013.10.008 24438723

[B4] Bigorra J. Gomis R. Sampietro-Colom L. Huc M. Lurigados C. Zamora A. (2009). “Desarollo de Un sistema de Conocimiento compartido para La evaluacion en red de La innovacion en red de La innovacion tecnologica en medicina,” in Informes de Evaluacion de Tecnologias sanitarias. La Evaluacion de Tecnologia En El hospital. (Madrid, Spain: Plan de Calidad para el Sistema Nacional de Salud. Ministerio de Ciencia e Innovación. Agéncia d'Avaluació de Tecnologia i Recerca Médiques de Cataluña), 15.

[B5] Bos M. (1995). “Health Care Technology in the Netherlands,” in Office of Technology Assessment, Health care technology and its Assessment in Eight Countries (Washington, DC: Government Printing Office), 171–208.

[B85] Chevalier F. Lévitan J. Garel P. (2009). Hospitals in the 27 Members States of the European Union. Paris, France: Dexia Edition. Available at: http://www.hope.be/wp-content/uploads/2015/11/79_2009_OTHER_Hospitals-in-27-Member-States-of-the-European-Union-eng.pdf.

[B6] De Pietro C. Camenzind P. Sturny I. Crivelli L. Edwards-Garavoglia S. Spranger A. (2015). Switzerland: health system review. Health Syst. Transit. 17 (4), 1–288. 26766626

[B7] EUnetHTA (2015). About EUnetHTA. Available at: https://www.eunethta.eu/about-eunethta/ (Accessed May 10, 2020).

[B8] Gulácsi L. Rotar A. M. Niewada M. Löblová O. Rencz F. Petrova G. (2014). Health technology assessment in Poland, the Czech Republic, Hungary, Romania and Bulgaria. Eur. J. Health Econ. 15 (Suppl. 1), 13. 10.1007/s10198-014-0590-8 24832832

[B9] GDP per capita (2018). PPP (current international $) – Switzerland. Available at: https://data.worldbank.org/indicator/NY.GDP.PCAP.PP.CD?locations=CH&name_desc=false (Accessed December 25, 2019).

[B10] Halmesmäki E. Pasternack I. Roine R. (2016). Hospital-based health technology assessment (HTA) in Finland: a case study on collaboration between hospitals and the national HTA unit. Health Res. Policy Syst. 14 (1), 25. 10.1186/s12961-016-0095-2 27044400 PMC4820927

[B11] HB-HTA (2020). Project prescription. Available at: https://hbhta.pl/o-projekcie/ (Accessed May 10, 2020).

[B12] INAHTA (2015). INAHTA What is Health Technology Assessment (HTA)?. Available at: Https://www.Inahta.Org/ (Accessed May 25, 2015).

[B13] Jivegard L. Ektroth R. (2007). “Single technology health technology assessment (Mini-HTA) by local health care professionals used as a local decision support tool in a Swedish health care region – a way to increase evidence-based care,” in Health Technology Assessment International Annual Scientific Conference, Barcelona.

[B14] Kidholm K. Ehlers L. Korsbek L. Kjærby R. Beck M. (2009). Assessment of the quality of mini-HTA. Int. J. Technol. Assess. Health Care 25 (1), 42–48. 10.1017/S0266462309090060 19126250

[B15] Kindholm K. (2019). Interview by Krzysztof Lach. interview online (Accessed September 10.2020).

[B16] Kisser A. Tüchler H. Erdös J. Wild C. (2016). Factors influencing coverage decisions on medical devices: a retrospective analysis of 78 medical device appraisals for the Austrian hospital benefit catalogue 2008–2015. Health Policy 120 (8), 903–912. 10.1016/j.healthpol.2016.06.007 27344197

[B17] Kowalska-Bobko I. (2017). Decentralizacja a systemy Zdrowotne. W poszukiwaniu Rozwiązań sprzyjających Zdrowiu. Kraków, Poland: Wydawnictwo Uniwesytetu Jagiellońskiego.

[B18] Lennart J. Bergh C. Kindblom J. Samuelsson O. Sjögren P. Sjövall H. (2017). “Activity-based HTA: hospital-based HTA performed by clinicians with support and quality control, the sahlgrenska university hospital HTA-centrum experience (Sweden),” in Hospital-based health technology assessment. The Next Frontier for health technology assessment. (Switzerland: ADIS Springer International Publishing).

[B19] Lipska I. McAuslane N. Leufkens H. Hövels A. (2017). A decade of health technology assessment in Poland. Int. J. Technol. Assess. Health Care 33 (3), 350–357. 10.1017/S0266462317000563 28720170

[B20] List of Countries by Projected GDP (2018). GDP Per Capita, PPP (Current International $) – Austria, Spain, Netherlands, France, Sweden, Finland, Denmark, Italy, Switzerland. Available at: https://data.worldbank.org/indicator/NY.GDP.PCAP.PP.CD?locations=AT-ES-NL-FR-SE-FI-DK-IT-CH (Accessed May 4, 2021).

[B21] Martelli N. Hansen P. van den Brink H. Boudard A. Cordonnier A.-L. Devaux C. (2016). Combining multi-criteria decision analysis and mini-health technology assessment: a funding decision-support tool for medical devices in a university hospital setting. J. Biomed. Inform. 59, 201–208. 10.1016/j.jbi.2015.12.002 26705065

[B22] Martelli N. (2019). Interview by Krzysztof Lach (Accessed September 10, 2020).

[B23] Sagan A. Panteli D. Borkowski W. Dmowski M. Domanski F. Czyzewski M. (2019). Poland health system review. Health Syst. Transit. 13 (1), 1–193. 22551527

[B24] Sampietro-Colom L. Martin J. (Editors) (2017). Hospital-based health technology assessment: the Next Frontier for health technology assessment (Cham, Switzerland: Springer).

[B25] Sampietro-Colom L. (2012). Consider context and stakeholders. Int. J. Technol. Assess. Health Care 28 (2), 166–167. 10.1017/S0266462312000153 22494695

[B26] Sampietro-Colom L. Lach K. Kidholm K. Cicchetti A. Fure B. Pasternack I. (2015). The AdHopHTAHandbook. Available at: http://www.adhophta.eu/sites/files/adhophta/media/adhophta_handbook_website.pdf (Accessed August 15, 2020).

[B27] Sampietro-Colom L. Morilla-Bachs I. Gutierrez-Moreno S. Gallo P. (2012). Development and test of a decision support tool for hospital health technology assessment. Int. J. Technol. Assess. Health Care 28 (4), 460–465. 10.1017/S0266462312000487 23062518

[B28] Wild C. (2019). Interview by Krzysztof Lach, interview online. (Accessed September 10, 2020).

[B29] Włodarczyk C. Kowalska I. Mokrzycka A. (2012). Szkice z Polityki Zdrowotnej Unii Europejskiej. Kraków, Poland: Wolters Kluver.

