# Supplementary material for: Recommendations for the Implementation of Hospital Based HTA in Poland: Lessons Learned From International Experience
Source: Front Pharmacol. 2021 May 13;11:594644. doi: 10.3389/fphar.2020.594644 (PMC8155722; doi:10.3389/fphar.2020.594644)
Supplement: Supplementary file 1 [file DataSheet1.PDF]

Appendix 1. Studies search strategy

1. Studies search strategy

Tab 6. Strategy of search for studies in MEDLINE (PubMed) base, cut-off date 2019/12/29.

	Key words	Results
#1	HB-HTA[tiab]	11
#2	Hospital-based Health Technology Assessment[tiab]	32
#3	"Hospital HTA"[tiab]	3
#4	"Local HTA"[tiab]	18
#5	"mini-HTA"[tiab]	19
#6	"mini HTA"[tiab]	19
**#7**	**#1 OR #2 OR #3 OR #4 OR #5 OR #6**	**60**

Tab 7. Strategy of search for studies in Embase base, cut-off date 2019/12/29.

	Key words	Results
#1	“HB-HTA”	24
#2	“Hospital-based Health Technology Assessment”	51
#3	"Hospital HTA"	5
#4	"Local HTA"	31
#5	"mini-HTA"	30
#6	"mini HTA"	30
**#7**	**#1 OR #2 OR #3 OR #4 OR #5 OR #6**	**108**

2. Studies search strategy

Tab 8. Strategy of search for studies in MEDLINE (PubMed) base, cut-off date 2019/12/29.

	Key words	Results
#1	HTA[tiab]	2,842
#2	"Health Technology Assessment"[tiab]	4,187
#3	"Technology Assessment"[tiab]	6,118
#4	#1 OR #2 OR #3	7,459
#5	interaction[tiab]	729,498
#6	interactions[tiab]	620,234
#7	collaborat*[tiab]	131,714
#8	cooperat*[tiab]	130,620
#9	co-operat*[tiab]	10,655

	Key words	Results
#10	joint[tiab]	248,101
#11	#5 OR #6 OR #7 OR #8 OR #9 OR #10	1,680,607
#12	#4 AND #11	739
**#13**	**#4 AND #11 Filters: published in the last 10 years**	**547**

Tab 9. Strategy of search for studies in Embase base, cut-off date 2019/12/29.

	Key words	Results
#1	HTA	8,710
#2	"Health Technology Assessment"	9,896
#3	"Technology Assessment"	29,223
#4	#1 OR #2 OR #3	34,144
#5	Interaction	1,461,246
#6	Interactions	712,289
#7	collaborat*	270,352
#8	cooperat*	273,873
#9	co-operat*	15,692
#10	Joint	603,160
#11	#5 OR #6 OR #7 OR #8 OR #9 OR #10	2,906,511
#12	#4 AND #11	3,860
**#13**	**#4 AND #11 AND [2009-2019]/py**	**2,718**

Appendix 2. Results of search strategies

Fig 1. Diagram of subsequent search and selection stages (PRISMA diagram) - search strategy (1)

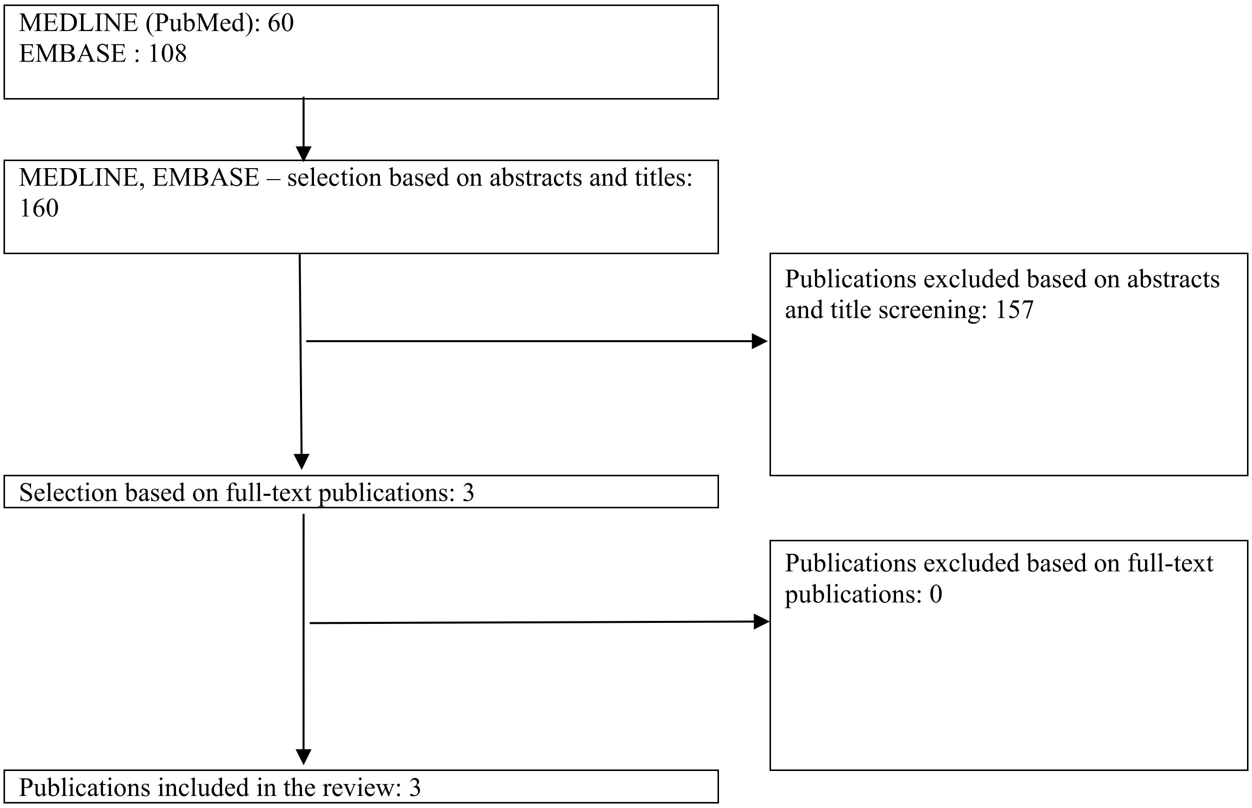

Fig 2. Diagram of subsequent search and selection stages (PRISMA diagram) - search strategy (2)

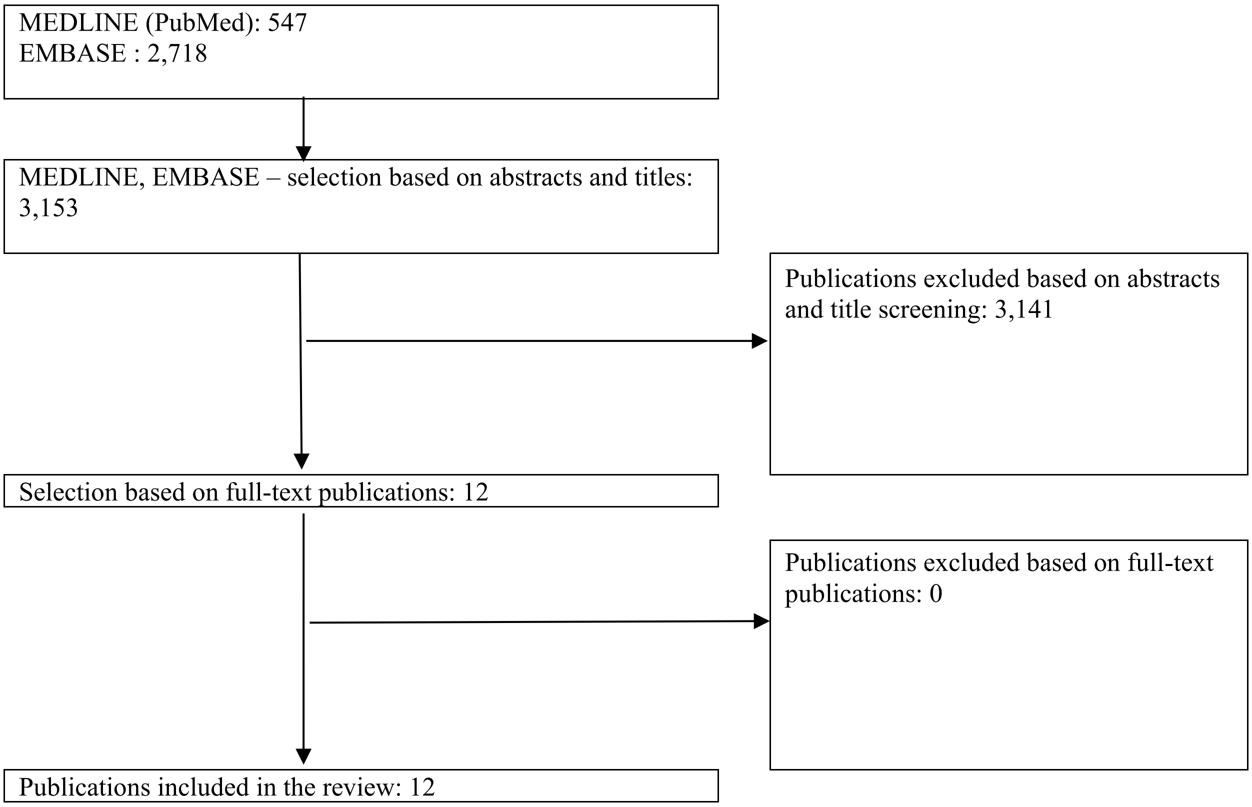

Appendix 3. HTA agencies characteristics in selected European countries

Country	HTA agency	Scope of responsibilities
**Switzerland**	Lack of HTA dedicated agency with stable formal status;<br>Federal Office of Public Health is responsible for health technology assessment.	• Inclusion of medical goods and services in the Mandatory Health Insurance benefit package;<br>• Decision-making process based on EBM;<br>• Costs rationalization;<br>• Informal institution Swiss Medical Board produce HTA reports and formulate recommendations for health providers and decision makers.
**Spain**	Seven autonomous regions HTA agencies (from Andalusia, Aragon, Basque Country, Catalonia, Galicia, Canary Island, Madrid)	• Introducing of new health technologies in the public health-care portfolio stays at the national level but final decision regarding introduction of medical devices is at the local or hospital level.
**France**	HAS (*Haute Authorite de Sante*).	• Launched in 2004;<br>• Scientific independent body and tasked with quality in health and social care;<br>• Cooperation with public authorities to report policy decisions as well as health professionals to optimize practices, organisations and services.
**Italy**	AGENAS (*The Italian National Agency for Regional Healthcare Services*)	• Funded in 1993;<br>• Supervised by the Ministry of Health;<br>• Responsible for healthcare services distribution.
**Denmark**	National Treatment Council	• Preparing mini-HTA economic assessment on the national level.
**Finland**	Finnish Health Technology Assessment Agency (*fin. FinoHTA*)	• Responsible for supporting and coordinating HTA reports as well as promoting multidisciplinary assessments;<br>• Involved in promoting the concept of HTA among clinicians and providing financial support for clinical studies such as randomized trials as well as acting as a national clearing house by collecting, analysing, synthesizing, and disseminating information on national and international HTA studies.
**Sweden**	Swedish Council on Health Technology Assessment in Health Care (*sw. SBU*)	• Reviewing the benefits, risks and costs of methods used in health care delivery, with the aim of identifying which method

Country	HTA agency	Scope of responsibilities
		is the most appropriate for treating a specific disease and patient group, but also to determine methods which are ineffective or not cost-effective, so that they can be rejected;<br>• Identifying important knowledge gaps in which further research is urgently needed.
**The Netherlands**	Health Care Institute (*nt. ZINL*)	• Responsible for the quality, accessibility and affordability of the healthcare system;<br>• Advising on and clarifying the contents of the standard health care benefit package;<br>• Assessment of outpatient medicines for the benefit of the Medicine Reimbursement System (GVS).
**Austria**	Ludwig Boltzmann Institute (*LBI-HTA*)	• Cost analysis;<br>• Actions harmonizing;<br>• Counselling for politicians and healthcare managers;<br>• Providing knowledge for other stakeholders.

Source: Own study based on review of professional literature, AdHopHTA project manual (online version) and medical information databases (PubMed, Embase).

Appendix 4. HB-HTA units characteristics in selected European countries

Country	HB-HTA Unit Name	HB-HTA Model / Staff	Source of financing and formalisation level	Scope of responsibilities	Stakeholders
**Switzerland**	HTA Unit (Lausanne);<br><br>Commission for new technologies plus specific committees for drugs and devices (Geneva);<br><br>Drug committee for	HTA Unit-integrated essential;<br>Health economist;<br><br>Commission for new technologies plus specific committees for drugs and devices-	Informal structure,<br>Donated internally from hospital budget,	• Conducting HB-HTA for management process;<br>• Introduction and use of new diagnostic or therapeutic procedures;<br>• Process is initiated by clinicians;	• Clinical hospitals (Lausanne, Geneva, North Vausdois);<br>• Medical Association and Health Sciences Association;<br>• Federal Office of Public Health.

Country	HB-HTA Unit Name	HB-HTA Model / Staff	Source of financing and formalisation level	Scope of responsibilities	Stakeholders
	drugs, Biomedical Engineering for equipment and devices (North Vaudois)	independent group; clinicians, Biomedical engineer,<br><br>Drug committee for drugs, Biomedical Engineering for equipment and devices; Health economist, external consultant form a university hospital		• Unit is in charge of budget impact and writing the HB-HTA report.	
**Spain**	HB-HTA Unit,<br><br>Joint Commission for HTA,<br><br>HTA Committee	HB-HTA Unit- stand alone;<br>medical doctor and public health scientist,<br><br>Joint Commission for HTA- integrated essential;<br>Internists, paediatricians, rehabilitation, thoracic surgeon, intensivist, pharmacist, biochemical specialists and public health and epidemiology specialist<br>HTA Committee- independent group;<br>head of quality, a psychiatrics expert in	Informal, financing from hospital budgets	• HB-HTA Unit- carries out HTA reports for the 11 clinical institutes of the hospital;<br>• Commission for HTA is in charge of approving or rejecting the proposed HTs that want to enter the hospitals;<br>• HTA Committee makes recommendations and brings them to the hospital steering committee for final decision;<br>• Common features from the three hospitals include origin of the request for assessment, funding of the unit/programme and	• Local governments of Spanish regions,<br>• Hospital Clinic Barcelona,<br>• Virgen del Racio & Virgen de la Macarena Hospitals;<br>• Hospital Sant Joan de Deu;<br>• Coordination of HB-HTA in Barcelona

Country	HB-HTA Unit Name	HB-HTA Model / Staff	Source of financing and formalisation level	Scope of responsibilities	Stakeholders
		health economics, the head of research, a nurse manager, a librarian and a surgeon.		enforcement of the recommendation's final decision;<br>• Merging clinical, economic and organisational information.	
**France**	Former Name: CEDIT;<br>Current Name: CODEINS	25 psychicians from different specialties, pharmacists, top managers, headquarter division directors; scientific secretariat hires people with diverse backgrounds: public health specialists, dentists, biostatisticians and biomedical engineers.	Informal, Internally and externally.	• Concentrated on medical devices and equipment, but covers all health technologies, including medicines, procedures, and even organisations, sometimes all at once;<br>• Embarked upon a "full HTA" by taking into account all the aspects of the assessment: technical, clinical, economic, and also "social acceptability" including organisational, ethical, and legal aspects.	HAS;<br>Clinical hospitals of Paris Region;<br>National Fund of Healthcare Insurance
**Italy**	HTA Innovation Unit;<br>Gemelli Hospital	Stand alone;<br>Biomedical engineer, health economist, statistician, pharmacist.	Statutorily regulated; formal structure, Financed from hospital budget.	• Proactive disinvestment process;<br>• Dissemination process;<br>• Budget impact analysis;<br>• Legal status research;<br>• Finding alternative solutions in literature.	• Gemelli Hospital (Technical Department and Controlling Department);<br>• Italian Medicines Agency (AIFA);<br>• Graduate School of Health Economy and Management;<br>• Ethics Department at the Universita Catholica del Sacro Cuore;

Country	HB-HTA Unit Name	HB-HTA Model / Staff	Source of financing and formalisation level	Scope of responsibilities	Stakeholders
**Denmark**	HTA Unit at Odense University Hospital;	Integrated specialized; Manager (health economist), two people with public health degrees, specialists in biomedical engineering;	Formal, external (sometimes financing from scientific grants) and internal (hospital management)	• Courses in mini-HTA;<br>• Assistance in the production of mini-HTA;<br>• Assistance in larger HTA projects.	• Department of Quality and Research inside the hospital;<br>• Region of Southern Denmark;<br>• Regional Treatment Board.
**Finland**	HTA Group Coordinated	Integrated specialized; Network of 65 clinicians	Formal, statutorily regulated, financed by regional funds	• creating a systematic review of the literature;<br>• rationalizing drugs' use; management<br>• clinical hospitals as innovators in introducing new technologies;<br>• cooperation between hospital HTA and HTA at the national level.	• FinoHTA;<br>• 15 hospital districts in the Helsinki Region.

Country	HB-HTA Unit Name	HB-HTA Model / Staff	Source of financing and formalisation level	Scope of responsibilities	Stakeholders
**Sweden**	HTA-Centrum	Integrated specialized; clinicians, health professionals, HTA professionals, medical librarians,	Informal;<br>Funded by external grants.	• Clinicians wishing to use new technology are responsible for producing an activity-based HTA;<br>• Technologies for activity-based HTA can be nominated by health-care professionals and managers;<br>• Supporting the nominated question and making work time available for the HTA to be conducted by the clinicians is the responsibility of the head of the department involved;<br>• In order to enable an on-demand activity-based HTA, a support organisation (HTA-centrum), including the Medical Library and a quality assurance process, is necessary;<br>• Literature research and article selection can be conducted mostly by medical librarians;<br>• Budget requests and research funding drive the activity-based HTA process.	• The Region Västra Götaland health-care organisation;<br>• Sahlgreska University;<br>• Medical Faculty of university.

Country	HB-HTA Unit Name	HB-HTA Model / Staff	Source of financing and formalisation level	Scope of responsibilities	Stakeholders
**The Netherlands**	HTA-Unit	specialized integrated;<br>health economists,<br>clinicians,<br>public health specialists	external funding from the national budget and internal hospital grants	• creating a public procurement council whose task is to analyse the cost of a given investment ex ante;<br>• Making decisions, better decision making;<br>• HTA decentralisation, beginning in hospitals;<br>• cooperating with other stakeholders;<br>• development of HTA methodology.	• Dutch Organisation for Research and Development;<br>• Radboud University Medical Centre in Nijmegen;<br>• National Health Insurance Board.
**Austria**	HB-HTA Unit in Tirol	Independent group;<br>public health professionals (workers in LBI-HTA and unit in Tirol),<br>clinicians (cardiologists and oncologists),<br>politicians,<br>regional representatives,<br>Hospital managers	Informal structure, funded externally from regional and LBI-HTA budget	• preparing assessment of very innovative health technologies; mainly rare disease;<br>• sharing HB-HTA reports;<br>• cost rationalization;<br>• providing correct reimbursement decisions and adding services to hospital catalogues;<br>• providing economic evaluation for drug and non-drug technology (mainly in oncology);<br>• support in disinvestment decisions.	• Ministry of Health;<br>• Austrian Social Security;<br>• Regional Hospital Cooperation;<br>• Health technology Assessment Committee;<br>• 9 Austrian districts/regions;

Source: Authors' own study based on review of professional literature, AdHopHTA Handbook and medical information databases (PubMed, Embase)
